# The Four Causes of ADHD: Aristotle in the Classroom

**DOI:** 10.3389/fpsyg.2017.00928

**Published:** 2017-06-09

**Authors:** Marino Pérez-Álvarez

**Affiliations:** Department of Psychology, University of OviedoOviedo, Spain

**Keywords:** accidental intolerance, ADHD, Aristotle’s four causes, Charcot effect, semiotic mediator

## Abstract

Attention-Deficit/Hyperactivity Disorder (ADHD) is one of the most well-established and at the same time controversial disorders to the extreme of being placed in doubt. In the first of two parts, the established position is critically reviewed, beginning with showing fallacious reasoning on which the diagnosis is based, lacking clinical proof. Similarly, a certain rhetoric and metaphysics in genetic and neurobiological research is highlighted, where, for example, a meager accumulation of data is offered as robust conclusions, and correlates and correlations as causes and bases. However, that may be, the controversy is silenced in a dialog of the deaf between “defenders” and “critics.” with no way out in sight in empirical and scientific terms. A new meta-scientific position is necessary to analyze the science of ADHD itself and its social uses. In this respect, the second part introduces Aristotle’s four causes, material, formal, efficient, final, as an instrument of enquiry. According to this analysis, ADHD is not the pretended clinical entity as presented, but a practical entity providing a variety of functions. The implications would be rather different from the usual.

## Introduction

This article takes a critical look at the established conception of what is called “Attention-Deficit/Hyperactivity Disorder" (ADHD). The established concept presents ADHD as a neurodevelopment disorder with a highly inheritable genetic origin which begins in infancy and frequently continues into adulthood. This concept forms part of the beginning of most articles on ADHD, as an already familiar *rhetoric* suggestive of something well-established by consensus.

However, the even authors arguing the standard concept recognize the controversy concerning its clinical entity, perhaps just another aspect of *its* rhetoric. The truth is that there is also extensive literature questioning the clinical medical-scientific validity of ADHD. The controversy may be reduced to two opposite positions: the standard, which states its well-established existence, such that denying it would be like denying that the Earth is round, and the critical, which denies its clinical entity, such that those who argue for it would only be pathologizing normal behaviors and problems. A third position, apparently between them, is limited to criticizing overdiagnosis and overmedication, but is still a variant of the standard concept.

Although the controversy is incessant, it does not seem to go any further in the usual terms of whether ADHD exists or not. The critical position cannot just deny its existence under the assumption that it is an “invention” of the pharmaceutical industry or the bio-power that be or whatever. Not because it is an invention would it no longer constitute a factual, practical, and institutional reality. The question would be what *does* exist. However, those who argue for ADHD cannot do so only with ambiguous rhetoric and questionable implicit assumptions. The question here would be *why* they are as genuinely convinced as they are of such a controversial position.

The controversy cannot be resolved in empirical scientific terms, on the plane of facts, as if the facts spoke for themselves, which is where it now stands. A metascientific, philosophical assessment is required, with an ontological scope asking *what* ADHD is, and epistemological scope asking *how* science itself knows and molds what has ended up as the actual “ADHD.”

This approach is based on a critical position (not ingenuous) concerning the impressive neuroscientific evidence claimed as support for the established concept. Such an approach is unthinkable for those who assume the standard concept, given their amazement that anyone would deny it. Without denying their data, light will be shed on the *rhetoric* and *metaphysics* that sustain it. If the rhetoric suggests more persuasive than truthful reasoning, metaphysics refers here to implicit assumptions about genetics and the brain which go *beyond* what genomics and brain connectomics really permit. The article has two parts. The first concentrates on revealing the rhetoric and metaphysics of ADHD neuroscience. The second develops the metascientific approach beyond the usual controversy – whether or not it exists – attempting instead to understand what it is that exists and how it came to be that way.

## Rhetoric and Metaphysics of the ADHD Neuroscience

Instead of uncritically assuming the standard ADHD concept as if scientific evidence required it, we review its consistency, focusing on the *rhetoric* and *metaphysics* on which it is largely based. Its three basic pillars, diagnosis, genetics, and neurobiology, are specifically reviewed.

### How to Make a Diagnosis with Fallacious Reasoning (with Tautologies)

Everything begins and has its basis in the diagnostic criteria of the Diagnostic and Statistical Manual of Mental Disorders (DSM; American Psychiatric Association, 2013). For the case in hand, it does not matter whether the International Classification of Diseases (ICD; World Health Organization, 1992) is used. It is the DSM/ICD diagnostic systems themselves which are questioned as a valid basis for making diagnoses. Suffice it to cite in this respect the critical position of two international psychiatric organizations. The first is the statement by Thomas Insel on April 29, 2013, as Director of the National Institute of Mental Health (NIMH), that the Institute was not going to use the DSM-5 criteria at the point of being released, as in fact it was in May the same year, due to its lack of validity. As Insel (2013) states:

The strength of each of the editions of DSM has been “reliability” – each edition has ensured that clinicians use the same terms in the same ways. The weakness is its lack of validity. Unlike our definitions of ischemic heart disease, lymphoma, or AIDS, the DSM diagnoses are based on a consensus about clusters of clinical symptoms, not any objective laboratory measure.

The Critical Psychiatry Network, an organization of critical psychiatrists who focus on a biomedical approach in psychiatry, has promoted a campaign under the slogan, “No more psychiatric labels” for abolition of the DSM/ICD, also on the basis of its lack of validity (Timimi, 2014).

The DSM may have improved reliability with regard to consistency among those who apply it, but validity is something else: its correctness and robustness for making a diagnosis. A Marcia Angell, ex-director of the *New England Journal of Medicine* says about psychiatric diagnoses:

If nearly all physicians agreed that freckles were a sign of cancer, the diagnosis would be “reliable,” but not valid. The problem with the DSM is that in all of its editions, it has simply reflected the opinions of its writers (Angell, 2011).

The diagnostic criteria are established by consensual opinion. But the consensus reveals that there is no evidence. If there had been scientific evidence, a consensus would not be necessary. What more can be said when many of the experts in the consensus are plagued with conflicts of interest and a laboratory in the field funds the meetings (Kooij et al., 2010)? These statements of consensus are upheld by the superabundant bibliography on ADHD, like someone who grabs hold of a lamppost to keep standing up without using it to see by. One consensus cites over 500 references (Barkley, 2002), like bulk evidence by accumulation, without reviewing it to see if it really is accumulated knowledge. Another uses 320 references to *back* its validity (Kooij et al., 2010), without really being clear, firm evidence. The statements of consensus assume a subtly deceitful argument consisting of citing quantities of studies, without any of them being conclusive, which in the end are taken as convergent, promising support. A collection of promising support turns into evidence. For example, after recognizing the challenge of a “diagnosis being based on reported symptoms alone; there are no biological tests” (Thapar and Cooper, 2016, p. 1241), ADHD becomes in the conclusions a “robust and consistent across design type and sample. There are established assessment methods” (Thapar and Cooper, 2016, p. 1247).

The truth is that the diagnosis of ADHD is established, but based on fallacious reasoning, typically two (Tait, 2009): the *Affirming the Consequent Fallacy* and *Begging the Question Fallacy*. According to the affirming the consequent fallacy, *if* the child “often fails to give close attention to details,” “often fidgets with or taps hands or squirms in seat”… (statements in the DSM-5 criteria) *then* he has ADHD. According to the begging the question fallacy it is already known that the child has ADHD because he/she “often fails to give close attention to details,” “often fidgets with or taps hands or squirms in seat,” and so on. The child does not pay attention and fidgets *because* he/she has ADHD and he/she *has* ADHD because he does not pay attention and fidgets. The symptoms are the guarantee of the diagnostic category, which in turn is invoked to explain the symptoms in an endless loop (Brinkmann, 2014b, p. 128). The diagnostic category is molded in the process of making the diagnosis of the case. In turn, the diagnosis of the case sustains the clinical category without other independent tests or genetic evidence or neurobiology of diagnostic value as discussed below. The *Non-Sequitur Fallacy*, which assumes that if the medication (typically stimulants) reduces hyperactivity, the hyperactivity is a symptom and ADHD is a disorder, also commonly occurs. But one does not follow the other, not logically and not empirically, since stimulants produce the same effect whether you *have* ADHD or *not*.

The diagnosis is based on fallacious reasoning, not on clinical tests as would be expected in view of how sure the affirmations are. Based on DSM/ICD diagnosis, a series of objective, complementary, and supposedly confirmatory tests are being studied. Among the most objective is the Test of Variables of Attention (TOVA), a continuous performance test (CPT) (Fried et al., 2014; Rodríguez et al., 2016). The TOVA presents a computerized task which evaluates omissions, commissions, reaction times, variability, and post-commission response times. Apart from the scant ecological validity as a task little representative of daily situations, the biggest problem with the TOVA is its inadequate specificity, leading to false positives and false negatives: children not ADHD who “fail” and ADHD children who do well (Zelnik et al., 2012; Fried et al., 2014).

The problem of ecological validity can be reduced with virtual reality tests. However, up to now support is limited (Neguţ et al., 2016). Virtual reality tests, even though they show sensitivity, also lack diagnostic specificity due to variability and overlapping of the measures (Areces et al., 2016; Neguţ et al., 2016). In fact, the TOVA, whether or not in virtual reality, is applied to children already differentiated by the DSM diagnostic criteria: children *with* ADHD or *without* ADHD, not as a diagnostic test itself. These tests can be useful for evaluating attention *skills*, precision or reaction times, which may be relevant in themselves without presupposing ADHD. They measure what they measure, but it could not be said that *the* ADHD is measured.

On the basis of the self-interested consensus that may be assumed from declared conflicts of interest and sponsorship of commissioned work (Barkley, 2002; Kooij et al., 2010; Faraone et al., 2015), inconsistent conclusions (Thapar and Cooper, 2016) and fallacious reasoning (Tait, 2009), surprisingly, or perhaps not, the sentence, as dogmatic as antiscientific, is that doubting ADHD would be like “declaring the Earth flat, the laws of gravity debatable, and the periodic table in chemistry a fraud” (Barkley, 2002, p. 90). According to Timimi et al. (2004) in his comment on this consensus, “It is regrettable that they wish to close down debate prematurely and in a way not becoming of academics. The evidence shows that the debate is far from over” (Timimi et al., 2004, p. 63). Even without assuming intentionality due to conflicts of interest in establishing a consensus, its unintentional influence through default brain-centered thinking cannot be discarded, as mentioned further below.

### How to Make Genetics Seem Like Evidence (with Ambiguities)

Beyond the diagnosis, one fundamental aspect of the ADHD rhetoric is its consideration as a highly inheritable genetic disorder (Tarver et al., 2014; Gallo and Posner, 2016; Thapar and Cooper, 2016). If the diagnosis is, as it is, based on a consensus dominated by conflicts of interest more than on tests, lacking in validity and more than anything else tautological, it is going to be hard for there to be specific genetic and neurobiological bases.

The reviews show two things: the non-existence of *real* molecular genetic evidence and the persistence in their affirmation. We refer here to reviews by authors who are not precisely critical of the genetic perspective, but as scientists are compelled to recognize what there is, not without their rhetoric. Thus Cortese, after reviewing the convergence of different approaches (none conclusive), refers to the future, “It is expected that future research will reveal similarly exciting convergent findings” (Cortese, 2012, p. 430). Thapar and Cooper (2016, p. 1242) recognize that “ADHD-associated genomic variants are non-specific.” A study by Thapar himself and others found that 14% of children diagnosed with ADHD had a rare chromosomic difference known as “copy number variants” compared to 7% of children not diagnosed with ADHD who also had it (Williams et al., 2010). Although the finding was magnified by emphasizing double the chromosome rarity in ADHD (13.95% over 7.4%) and was even taken as “direct evidence that ADHD is a genetic disorder” (Wellcome Trust, 2010), the truth is that 86% of children with ADHD did not have this rarity and 7% of those without ADHD did.

Tarver et al. (2014, p. 763) likewise acknowledge that “Genomic-wide searches have yet to identify a single candidate gene,” although they add that “This is probably due to insufficient sample sizes to date.” However, as suggested by Sonuga-Barke (2010, p. 113), “We are now using larger and larger samples of patients to demonstrate smaller and smaller molecular genetic main effects.” As Thapar et al. (2013) admit in their conclusions:

The genetic risks implicated in ADHD generally tend to have small effect sizes or be rare and often increase risk of many other types of psychopathology. Thus, they cannot be used for prediction, genetic testing or diagnostic purposes beyond what is predicted by a family history” (Thapar et al., 2013, p. 3).

Gallo and Posner (2016) also recognize the scant genetic evidence, but not without inconsistent rhetoric between what they affirm and what they really find. They state that “ADHD is a highly heritable disorder” (Gallo and Posner, 2016, p. 558) to continue by saying, “Despite substantial evidence for a genetic origin of ADHD, specific genes or sets of genes causally linked to the disorder have yet to be discovered” (p. 559). This rhetoric of inconsistencies continues when they say, “Substantial progress has been made in clarifying the complex genetic architecture of ADHD, yet the mismatch between the high heritability estimates and weak associations between ADHD and specific genetic markers is puzzling” (p. 560). After all, they feel obliged to admit that “Although candidate genes and neuro transmitter systems have been implicated in ADHD, genome-wide associations between ADHD and individual genetic variants have yet to be found” (p. 563).

The claimed heritability in ADHD is based on statistical data, not on *genetic* data as such. It often refers to ADHD running in the family and to the higher coincidence in monozygotic than in dizygotic twins. Many things run in the family, such as an accent in language or religion without therefore being genetic. Identical twins share more environmental conditions than non-identical twins so this and other “twin method” reasons do not enable the genetic-environmental knot to be unraveled, much less talk about percentages (Joseph, 2015).

Attention-Deficit/Hyperactivity Disorder may be hereditary, but not therefore genetic. Of the four ways of inheritance, genetic, epigenetic, behavioral, and cultural (Jablonka and Lamb, 2005), genetic is probably the least expectable in transmitting ADHD-type behavioral traits. It is not among the functions of genes to generate behavioral traits. The metaphors “code” and “program” have seduced the imagination of scientists, professionals and people in general so they sound like genes do more than they really do. As development *resources*, more than deterministic “programs,” everything related to genes depends on the context, from cellular, extracellular, embryonal, and intrauterine to perinatal and social, from womb to tomb. Recent research in epigenetic and genomic plasticity causes genes to be reconceived beyond their traditional conception as agents instructing traits (González-Pardo and Pérez Álvarez, 2013). The new conception means a change in genes to genome (including non-codifying matter) and of action to reaction referred to genome reactivity as a dynamic system “exquisitely sensitive” to signals in both the organism’s immediate intracellular context and external environment as a whole (Keller, 2014, p. 2428). The genome *mediates* adaptation and response to the environment; it does not *cause* response and adaptive action. As Keller (2014, p. 2427) says:

In addition to providing information required for building and maintaining an organism, the genome also provides a vast amount of information enabling it to adapt and respond to the environment in which it finds itself.

For lack of firm evidence, the defense of ADHD genetics at all costs is served by rhetoric, with two formulas, one consisting of saying that ADHD is a “heterogeneous,” “multifactorial,” or “complex” disorder, and the other emphasizing “gene–environment interaction.”

Talking about “heterogeneous,” “multifactorial,” or “complex” in psychiatry is synonymous today with lack of specific genetic evidence (Joseph, 2009, p. 72). These expressions sneakily suggest the genetic condition of a disorder by implying a complicated involvement of numerous genes, with no more evidence than thin correlational associations. But an association is not causation. For lack of precise and specific evidence, the genetic argument of ADHD becomes a loop. As Pittelli (2002) says when commenting on a meta-analysis, “The argument that ADHD is “mediated by many genes acting in concert” is rather circular in that it is based primarily on the complete failure of molecular genetic studies to find such genes and replicate those findings” (Pittelli, 2002). ADHD is still “complex” even without thinking about genes.

The commonly referred to gene–environment interaction also insinuates that there is more than there really is: supposed genes interacting with the environment. The formula here is sibylline, as deceitful as it is hard to contradict. Nevertheless, two arguments must be considered.

In the first place, if the ADHD genes are not identified, and they are not, then it is going to be hard to talk sensibly about their interaction. What are we talking about when we discuss interaction? Are the genes active agents able to interact following a “program” or “code” for some behavioral trait? What specific interactions are in play if not even the ADHD phenotype is well-defined? Endophenotypes supposedly closer to genetic influences, such as reaction times, response inhibition or working memory, are also discussed. But research shows that endophenotypes lack specificity for the ADHD phenotype itself (Gallo and Posner, 2016). Endophenotypes are expected to “increase statistical power to identify relevant associations between genes and neurobiological mechanisms,” but they remain “a promising route” (Gallo and Posner, 2016, p. 560); the new promise, not cold hard findings.

The second argument says the gene–environment or biology-culture dichotomy itself loses sense in genomic times (Keller, 2012, 2014). It no longer makes sense to talk about interaction as of two preexisting things that enter into interaction (gene–environment) and much less percentages of heritability.

What research in genomics has shown is that biology itself is constituted by those interactions, and is so constituted at every level, even at the level of genetics. Indeed, one might say that what makes a molecule—any molecule—biological is precisely its capacity to sense and react to its environment (Keller, 2012, p. 139).

The reactivity inherent in biological systems enables development to be understood as a set of cells with their multiple molecules, functioning in mutual concert for a certain result, not thereby executing a central program (Fisher et al., 2011, p. 74). According to this conception of development, it is hard to argue the conception of genes as “instructor agents” or “instructions” of traits which some day, for example, at school age, or in adult life, will be “expressed” as ADHD behavior. What persistent genetic research really underlines is the decisive role of environment in the development of mental disorders (Sonuga-Barke, 2010). It no longer makes sense to talk about percentages. Nature itself, constituting organisms in their continuous interactions, places us beyond typical topical percentages.

The tangled nature of genetics and the environment, even in genetic conditions such as diabetes, keeps heritability from being unpacked, or from there being any interest in doing so (Chaufan, 2008). To begin with, heritability in its technical sense is an attribute of a population, not of individual traits. Furthermore, organisms and phenotypes are *non-additive* products of genes, in an historic sequence of development environments and chance events, so their interdependence impedes any empirical or statistical quantification of the “ingredients” in this mixture. Empirically, the genetic-environmental percentage could be established in breeding animals and in agriculture, from which statistical techniques are derived, enabling quantification that makes sense under controlled conditions. But statistical analyses (typically analysis of variance) are not analyses of *causes*, and therefore do not permit understanding what *caused* a disorder in an individual (Chaufan, 2008, pp. 21, 35). The problem of heritability is not resolved with *larger* samples (Chaufan, 2008, p. 37), as in the promise of the overused genetic perspective (Thapar et al., 2013, p. 7; Tarver et al., 2014, p. 763; Gallo and Posner, 2016, pp. 559–560). It has already seen in schizophrenia, a clinically well-established disorder (Pérez-Álvarez et al., 2016), unlike ADHD, how larger samples of thousands of patients do not lead to stronger genetic associations (Ross, 2016; Sekar et al., 2016).

While the concept of hereditability is confusing at least, for understanding the category, it lacks application to individuals. Even if there were “ADHD genes,” *pre*disposition does not imply availability of the phenotype, according to epigenetic chance (González-Pardo and Pérez Álvarez, 2013; Dillon and Craven, 2014; Mukherjee, 2016).

Quantification of heritability has little to offer for understanding ADHD. According to Chaufan (2008), the genetic emphasis may even be harmful to the extent that it diverts public attention and research funding from the social determinants, which are decisive in the end, even in genetic conditions such as diabetes. There may be more science policy than science itself in pursuance of genetics, concerning interests and status of the authors involved, beginning with the hegemony of the biomedical model.

### How to Make Causes Out of Correlations and Correlates (by Calling Them “Bases”)

A critical position must also be adopted regarding ADHD as a disorder of neurodevelopment, as presented in its standard packaging, instead of simply assuming it without further consideration. The literature in favor of the neurodevelopment approach is undeniably enormous and the amount of data overwhelming. However, the data do not speak for themselves, but by the perspective in which they are taken. In an uncritical neurodevelopment perspective which places the brain under spotlights as if it were the place the keys to ADHD should be found, data providing feedback for this search are not lacking nor will they be. Things always happen in the brain related to the activities of organisms. It would be of concern if it were not. As more and more sensitive measurements of the brain’s functioning become available, neural correlates of the activities selected are found more easily. Another thing is the relevance of the findings and the meaning of the correlation: causal and in what direction, or artefactual due to third factors involved.

A brain-centered approach like the one predominating research and propagated for ADHD incurs easily in two biases: a *tunnel effect* in which one looks in only one direction and a *zoom effect* which magnifies what is seen. A new panoramic and even telescopic approach is necessary which puts the brain in its place: in the body of a subject who behaves within a context and who sees from a distance what is known without becoming “stuck” to the data. Without doubt, more and more is known about the brain due to the new technologies and concepts of its functioning, but not because of this is more known about ADHD, as it has been demonstrated that in spite of everything, its status is still controversial. There is a mountain of data, that is, an enormous amount has been accumulated, but it cannot be said that it is really the solid, accumulative scientific knowledge with which research progresses. After all, there are no “diagnostic neurobiological markers” (Thapar and Cooper, 2016, p. 1243), the “underlying mechanisms” are unknown (Cortese, 2012, p. 2) and in general, “findings from neurobiological research do not have a direct application in daily clinical practice,” (Cortese, 2012, p. 9).

The variability and inconsistency of the findings may be reflecting the heterogeneity and lack of entity of the so-called “ADHD.” As Beare et al. (2016) say of their own findings:

Attention-Deficit/Hyperactivity Disorder is an extremely heterogeneous disorder, with few common findings across studies. The variability in findings resulting from methodological decisions in this study illustrates the caution that must be taken in relating network differences to underlying neurobiology.

Neurobiological research is moving from focusing on brain areas toward dysfunctions in circuits distributed throughout the brain, leading to the new concept of “pathoconnectomics” (Cao et al., 2015). Pathoconnectomics assumes that major psychiatric disorders (e.g., ADHD) involve abnormalities of brain networks and that understanding the aberrant organization of brain networks is critical for understanding these brain disorders (Cao et al., 2015, p. 2802). Connectomics combines the study of structural connectivity between regions and functional connectivity consisting of synchronies of remote neuronal activities (Cao et al., 2015). A set of sophisticated mathematical techniques and functional magnetic resonance imaging along with the more conventional electroencephalography/hemoencephalography and infrared spectroscopy, enable a new topology and cartography of the brain (Rodríguez et al., 2011; Cao et al., 2015).

Both regional and general wiring impairments have been found in ADHD. Among the regional impairments, are volumetric reductions in the basal ganglia and abnormalities in cortical thickness in frontal and parietotemporal brain regions (Gallo and Posner, 2016, p. 556). In functional connectivity, one “of the more commonly reported abnormalities is reduced connectivity within the default mode network (DMN)” (Gallo and Posner, 2016, p. 557). DMN is a set of areas in the brain described in 2001 which is associated with mental processes at rest or wandering when one is not busy in some concrete external task (Raichle, 2015).

The hypothesis is that persistent activity of the DMN can interfere with the cognitive control network (CCN) involved in executive functions. Another hypothesis is that individuals with ADHD may have weaker connectivity in DMN when at rest, suggesting immaturity and atypical development. The two networks, DMN/CCN, seem to work in opposite directions in attentional tasks, “As attentional demands increase, activation of the CCN increases, whereas DMN activation decreases; conversely, during periods of internally focused cognitions, activation in the CCN is reduced, and DMN activation increases” (Gallo and Posner, 2016, p. 558). Interplay of correlative transitions would mean normal, mature and typical development.

Four considerations must be made before or instead of taking the neurodevelopment brain-centered perspective automatically, by default, as if there were no other option.

In the first place, the neurodevelopment approach leads to establishing a dichotomy between typical, normal or mature development and atypical, abnormal or immature development defining a psychiatric disorder (Cao et al., 2015). This dichotomy could be induced by the logic of the approach itself more than anything else, with its tunnel and zoom effects, selecting and magnifying some things and leaving others out. If individuals are selected for certain more or less conspicuous characteristics and taken to extremes with respect to others who do not have them, more or less associated neuronal correlates could appear depending on how well defined those characteristics are. Take the conditions of being a taxi driver or a non-taxi driver, or a musician or non-musician. As studies show, the brains of taxi drivers and musicians show alterations in specific areas and connections associated with their activities compared to those who are not (Maguire et al., 2006; Hyde et al., 2009). Now it could be said that taxi drivers and musicians develop an “atypical” brain compared to non-taxi drivers and non-musicians, who after all, make up the majority of the population, and would develop a “typical” brain. There might also be subtypes: Subtype pianist and subtype violinist and who knows whether subtypes of London, Bombay or small-town taxi drivers.

In fact, as a second consideration, the supposed “neuronal bases” for ADHD do not consist of anything but correlations and correlates. They are “associations,” which however, implicitly suggest neuronal causes as “bases” (Rubia et al., 2014, p. 532), when not explicitly (Gallo and Posner, 2016; Rodríguez et al., 2016). Thus, Gallo and Posner (2016), after warning about the “limitations of correlational research” (p. 561) and “caution in imputing causality” (p. 563), in the end are thinking about mapping “causal pathways from genes to neural circuits to symptoms” (p. 564). Rodríguez et al. (2016, p. 8), on the other hand, argue for a “causal model” based on explicit assumptions required by the “structural equation modeling” which they use when they refer to the “direct effect” of cortex activation of variables measured by the TOVA as an ADHD index.

As a third consideration, in a discussion of causal directions, it would be more coherent, both in conceptual terms based on brain plasticity and empirical terms related to correlations and correlates found, to argue for the opposite hypothesis, that behaviors themselves are the causes of the correlates or neuron “alterations” found. It would be more coherent to understand cerebral variations as “dependent variables” of the activities that organisms carry out in their environment than as causal “independent variables.” The brain changes and adapts in line with an organism’s activities depending on the requirements of the environment. The neuron correlates or brain “alterations” of the taxi drivers and musicians are *not* the cause of driving taxis or playing the piano or the violin. The explanation which Rodríguez et al. (2011) themselves offer in the case of measuring blood flow activated in the brain by cognitive tasks and educational exercises is coherent with this causal direction. After subjects are instructed to make mental calculations, oxygenated hemoglobin in their blood increases. As the authors say:

This approach, combined with educational exercises as *brain-training*, can maximize blood oxygenation directly in certain areas of the brain (Rodríguez et al., 2011, p. 66).

Beyond the ADHD cortex causal model assumed by “structural equations” (Rodríguez et al., 2016), the *real* causation seems to be from the behaviors to the brain (Rodríguez et al., 2011).

*Convergent* evidence, to use the rhetoric in vogue, is found in the abundant literature showing that ADHD behavior can be modified by exercises and behavioral training, which is hardly understandable if it has a neurological cause and genetic origin. Furthermore, normalization of the brain, which is usually referred to after medication with stimulants (Rubia et al., 2014, p. 529), could be due to its effect on behavior, and it would really be the change in behavior that is promoting change in the brain, something suggested by the authors themselves when they cite a study in which 4 weeks of training in juggling induced relevant changes in the brain (Rubia et al., 2014, p. 529).

A last consideration concerns how the neurodevelopment model reflects the problem of ADHD in real life in the *brain space*, both scientific related to its entity, and ethical referring to the evaluation involved. Thus the reviews still warn of continuous ambiguities and inconsistencies, no matter how interested they are in finding accumulated knowledge (Rubia et al., 2014, p. 523; Gallo and Posner, 2016, pp. 558, 560). These neurobiological ambiguities and inconsistencies probably reflect the very heterogeneity of “ADHD.”

The brain space also reflects ethical evaluation of ADHD, describing the “findings” as volumetric or connectivity “reductions,” white matter “deficits,” “retarded” maturity, “abnormality” or “atypical” development. Transferring normative values to the brain incurs in three problems. First, components which are no more than normative values are neutralized as natural. Second, maturity is linked to age and environment in a disease. Finally, maturity itself is misunderstood as an autonomous process independent of the setting, ignoring that “maturation” is not merely a question of age, but also of what occurs during one’s life.

## Conclusion

A critical review of the standard conception of ADHD has underlined the tautological (rhetorical) reasoning and assumptions implicit in the causal-explanatory role of the genome and the brain (metaphysics) which impregnate it. It is understood that this conception cannot be taken uncritically as a starting point. Although this review may seem “demolishing,” it is not everything. Up to here the criticism has been *negative* (“demolishing”), not reconstructive (explanatory) of what there is. After all, ADHD does *exist*. It is *real*. Negationist critics must recognize that ADHD does *exist*, since we are even discussing it, though it may be to argue and deny it. The question now is to see *what it is that exists, the way it is real*.

In this respect, we recur to an ontological metascientific and metatheoretical approach, beyond the facts and terms of the controversy itself. A new *radical* approach of this type related to the fundamental nature of something, and *whole* considering the different sides and dimensions of the problem, is found in Aristotle’s four causes.

## Metascience of ADHD: Aristotle’S Four Causes

Aristotle’s four causes do not refer to empirical or scientific causes. The terms Aristotle uses in his *Physics and Metaphysics*, in which he deals with causes are *aition*, in plural *aitia*, which is where *etiology* comes from. *Aition* has a wider sense than *cause* in English or Spanish. Aristotle’s causes refer to explanatory factors or principles that approach “why-questions” in order to explain why something exists the way it is (van Fraassen, 1980, p. 42). In any case, “cause” remains the best term to capture the meaning of Aristotle (Guthrie, 1981, p. 223). In the 21st century, we can still find refreshing thought in Aristotle for the problems of our times. In particular, the doctrine of the four causes is still useful in the “sublunary world” of human things, so the process of construction (workable materials, shapes, agents, purposes) is not lost from sight and thus does not fall or remain in mechanicist causes. The main Aristotelian causes of why, more than mere empirical causes, enable us to think about how science itself works, from a metascientific perspective.

Application of the four causes is not common in psychology, but neither is it unheard of (Killeen, 2001, 2004; Pérez-Álvarez et al., 2008; Pérez-Álvarez, 2009; Ribes-Iñesta, 2015). In the clinic, the *material cause* asks what psychological disorders are made of, the *formal cause* asks what shape they take, why they are that way, *the efficient cause* asks who makes them that way, and the *final cause* asks what purpose they have or what they are for (Pérez-Álvarez et al., 2008). Even though the typical examples of Aristotle’s efficient cause are actors or individual makers (sculptor, potter), here the possibility of collective actors or institutional agents (school, family, clinic) is also considered.

The four causes have been specifically applied to ADHD (Killeen et al., 2012). In spite of being systematic, documented, and well-argued, the application by Killeen et al. (2012) failed in important respects. To begin with, it fails by not fitting better to Aristotle’s original concept, which would have made it of greater interest. In the end, their application ends up being a mere reorganization of data from the official neurobiological concept, and ignores that this concept itself is in question.

The greatest contribution of the four causes might be in reconsidering the fundamental question of the way in which ADHD exists. In this article, the application by Killeen et al. (2012) will be briefly discussed first. In continuation, a new, more appropriately Aristotelian application is proposed to find consequences that could reorient the controversy and eventually overcome it.

### Causes without Revelation or Rebellion: Aristotle for Nothing

Killeen et al. (2012) applied the four causes in the following order: formal, efficient, material and final, distinguishing in turn, according to Aristotle, close or molecular (proximate) and ultimate or molar (distal) causation. The proximate formal cause of ADHD would be the *formal* DSM/ICD diagnosis itself. The ultimate formal cause would be given by the explanatory theories, typically in terms of executive functions. The proximate efficient cause would consist, according to Killeen et al. (2012), of the symptom triggers. They refer to inadequate reinforcement, processing demand overload (speed, duration, complexity), inadequate control of context (chaotic, stressful, unpredictable), boring environments and repetitive tasks. The ultimate efficient cause refers to the origins of the prenatal syndrome (maternal smoking, alcohol) and perinatal (head injury, malnutrition, stressful environment).

The proximate material cause, according to Killeen et al. (2012), would have to do with neurophysiological substrates, dynamic brain events and neuromodulatory systems. The ultimate material cause concerns genetic and epigenetic conditions, static brain structure and differences in the brain. The final proximate cause was found by Killeen et al. (2012) in negative reinforcement (escape from boredom and escape from mental fatigue) and positive reinforcement (approach novel stimuli, achieve goals more quickly, peer approval). The final ultimate cause would be in evolutionary usefulness referring to historical environmental consequences (new niches) and adaptive advantages (exploitation of opportunities, escape from stressful environments).

The application of Killeen et al. (2012) has several problems, beginning with the order of the causes: formal, efficient, material and final. The order is not indifferent, because it determines the interplay and scope of the causes. The logical, chronological and definitively, ontological would be material, formal, efficient and final as usually expressed. The material and formal causes go first, are interdependent on each other and imply the role of the others. Even when Aristotle gave the most importance to the formal cause as form, *eidos*, pattern, that which defines something as what it is, whether a statue or a bowl, the material as amorphous, unshaped raw material comes first, the marble in the statue or the clay in the bowl.

To begin with, the formal cause, which in ADHD, we could agree with Killeen et al. (2012) is the diagnosis made, means attributing the diagnosis entity in its own right, when the diagnosis itself is in question. Without further questioning, the rest of the causes revolve around the diagnosis, with all its assumptions, as if we were discussing a well-established clinical entity. Thus, the proximate efficient cause then becomes a mere trigger of ADHD symptoms as if it were a natural entity. The distal efficient cause would be in the perinatal antecedents. But the true Aristotelian sense of efficient cause refers to “actors” (individuals or groups) not “factors” or “triggers.” The notion of antecedent event does not capture the sense of efficient cause as the builder who builds.

Killeen et al. (2012) found the material cause in the neurophysiological substrates (proximate cause) and in supposedly genetic proneness (distal cause). Nevertheless, this material cause is neither justified by scientific evidence (according to the discussion in the first part) nor is it homogeneous with regard to the formal cause. While the diagnostic form of ADHD is defined by behavior on a molar scale, the material refers here to neurophysiological substrates on a molecular scale, a leap of scale also taken with regard to the efficient cause. While the efficient cause, according to Aristotle is on the operatory scale due to agents, authors or “actors” (not “factors” or “triggers”), the material cause of Killeen et al. (2012) is on the physicochemical molecular scale. As they themselves admit, the problems met with are hyperactivity and inattention (behaviors), not with “ADHD” (Killeen et al., 2012, p. 415) or in this case, the neurophysiological substrates. The sculptor and potter work with marble and clay as workable, moldable materials, not their atomic molecular substrate, which some may be.

Concerning the final cause, the sense of Killeen et al. (2012) as positive and negative reinforcement may be assumed. However, the ADHD fabric has other actors in addition to the person diagnosed, involving a complex of final causes.

In the end, the application of Killeen et al. (2012) simply reorganizes the data in a certain way, without suggesting their ontological status, scientific epistemology, social practice or political ethics of a complex phenomenon with numerous actors and interests, not without reason controversial. The causes of Killeen et al. (2012) are causes which neither reveal nor rebel. Aristotle’s four causes duly applied could reveal the scientific-practical tangle with which most convictions and best intentions sustain ADHD even though lacking in clinical entity and thus having grounds for a rebellion with cause.

### Rebellion with Causes: Unmasking the ADHD Tangle

The four Aristotelian causes are related to each other in such a way that it is practically impossible to discuss one without assuming the others. However, for analytical and explanatory reasons, it is advisable to go one by one. Specifically, it is important to begin with the material cause. Not in vain, the material cause is the *raw material* from which something is made (the marble in a statue or the clay in a bowl).

#### Material Cause

The material cause of ADHD would be the behaviors by which, in fact, it is defined. It refers to some behaviors of children with problems in certain tasks and in certain school, family, and social contexts. These behaviors, typically inattention, hyperactivity or impulsivity, become conspicuous and end up by defining a syndrome and the child itself, but in themselves are not problematic or pathological (“symptoms”), nor do they exhaust what the child is. Such behaviors attract attention and become problematic in terms of norms and values (Hawthorne, 2010; Brinkmann, 2016). But they are not symptoms of any disease, such as a seizure in epilepsy, trembling hands in Parkinson’s or the loss of memory in Alzheimer’s. They are part of a person’s *comportment* which is not reduced to a few behaviors.

A somewhat problematic distinction in the English language between comportment and behavior is introduced. In contrast to the term behavior that usually only captures discrete aspects of the person, typically symptoms, as is the case in ADHD, comportment refers to the whole Gestalt of being engaged with the world. The concept of comportment introduced here captures the “*unifying structure of embodied affective (and cognitive) engagement with the world*, as the most general term to refer to all-encompassing changes,” (Jacobs et al., 2015, p. 90). Comportment establishes our *constitutive relationship* with the world. This does not refer to an organism or individual separate from a world they interact with, but a mutually constitutive relationship in which comportment, specifiable in behaviors for practical reasons, is the *soul* and *incarnation* of this relationship.

The structure of comportment constitutes a situated functional corporal unit (Merleau-Ponty, 1942/1963; Thompson, 2007, p. 67). How we are situated is characterized by a phenomenical structure (perceptive and operatory) *from-toward, from* what we pay attention to *toward* something and then we operate *on* it. The human biophysical structure itself propels both forward and outward, opening way on a horizon of time and space. As sentient subjects and agents we are embodied, embedded, and enacted subjects (Thompson, 2007; Fuchs, 2011). Belonging to the world in this way means that the essential way we relate to things is neither purely sensory and reflexive, nor cognitive and intellectual, but bodily and practical, articulated by “motor intentionality.” This bodily motor intentionality-environment loop constitutes what Merleau-Ponty calls the “intentional arch,” which subtends our relationship with the world integrating sensitivity and motility, perception and action (Merleau-Ponty, 1945/1962, p. 136).

In this phenomenological, existential and behavioral perspective, behaviors, including those defining ADHD, are understood according to a circular causality or functional cycles of perception and movement: interplay, feedback or reinforcement. Three cycles have been described (Fuchs, 2011): *cycles of organismic self-regulation* engendering a basic bodily sense of self; *cycles of sensorimotor coupling* between organism and environment, and *cycles of intersubjective interaction*. The problems come up when the functional cycles are somehow altered, but would not therefore be diseases *of* the brain (Fuchs, 2011, 2012).

The behaviors by which ADHD is defined begin to attract attention and even become problematic because they alter functional cycles, starting with the intersubjective interaction cycles. A phenomenological study done with ADHD adults highlighted a certain experience of time and rhythm characterized by a desynchronized way of being-in-the-world (Nielsen, 2016). This desynchronization refers to an accelerated rhythm in thinking, bodily discomfort and even anxiety in movement that is not in time with the rhythms of others, of things, of places or of events. An analysis of the different rhythms of daily life (Lefebvre, 2004) would probably explain many things before pathologizing the different rhythms and styles.

In brief, the material cause ADHD is made of would consist of certain behaviors by which in fact it is diagnosed. Within this, it has been attempted to show that the behaviors forming part of functional cycles constitute the material itself of psychological problems (Pérez-Álvarez et al., 2008; Fuchs, 2012), including so-called ADHD, in which the problem would be a certain desynchronized way of being in the world (Nielsen, 2016).

#### Formal Cause

The formal cause of ADHD would be the *formal* diagnosis itself made by the diagnostic systems in use (DSM/ICD), in agreement with Killeen et al. (2012). It is no longer important that the diagnosis is more than anything tautological and lacking in validity, as discussed above. The diagnosis ends up by becoming objectivized and obvious, through the process of selection, definition and magnification of some behaviors over others, appropriately converted into “symptoms.” ADHD as it is in common use in school, family, and clinical contexts, as well as in the media, functions as a model, form or “cultural idiom.” A cultural idiom is made up of value systems, ways of interpreting, and epistemological assumptions, all of which structure the way in which people experience, give meaning to, and react to the situations they face (Vanthuyne, 2003).

The formal cause includes the theoretical models proposed for explaining ADHD behaviors (Killeen et al., 2012). Among the variety of models existing (Kofler et al., 2016) are those which postulate impaired executive functioning, such as behavioral inhibition (Barkley, 1997) or monitoring attention (Brown, 2005). Within their different emphases, they coincide in understanding the breakdown in executive functioning as some type of disruption in the brain. The attractiveness of an explanation in terms of executive functions may be in the apparent description of neurocognitive mechanisms which supposedly account for the behavior of individuals, something doubtless very much in agreement with the individualist, neuroscientific and biomedical view of our times. However, in spite of all their neuroscientific sophistication, the notion of executive function is still a mechanicist, homunculist explanation, by personifying in an internal *Cartesian* scenario what in fact individuals are doing in the real world scenario where they *execute* their life. After all, neither the intentions are given any place in the brain (Schurger and Uithol, 2015), nor is the supposed breakdown in executive functioning found everywhere (Brinkmann, 2016; Kofler et al., 2016).

The mechanisms which theoreticians and users of executive functions *hypostasize* as if they had a will of their own are only elements in a wider system in the sense of the structure of comportment mentioned above (Merleau-Ponty, 1942/1963) and its patterns of bodily interaction with the environment (Maiese, 2012). In its reconsideration of “central executive,” Michelle Maiese emphasizes the essential role of the *affective framework* in which “we interpret persons, objects, facts, states of affairs, and situations in terms of *embodied desiderative feelings.*” “Such framing typically occurs during essentially embodied, spontaneous subjective experience, prior to conceptual and propositional information processing, and yields a pre-reflective, non-conceptual, fine-grained contouring of that world, so that we immediately can target and focus our attention” (Maiese, 2012, p. 901).

There may be different forms of bodily harmony with the surrounding world among individuals and situations and even within the same individual depending on what attracts their attention and interests them. This would place the differences among individuals and within the individual himself more within an affective framework than neurocognitive abstracts of a central executive function. In fact, those who are ADHD are *not* ADHD all of the time nor everywhere, any more than by diagnostic *prescription.* Particular attunement to the environment, rather than a general breakdown, seems to be the problem of ADHD.

In short, the formal cause of ADHD would consist of the diagnosis itself which provides it with entity in its own right. The diagnosis already functions as a “cultural idiom” and counts on theoretical models which support it. It has been attempted to show that the overused model based on executive functions, far from explaining the supposed breakdown, returns ADHD to its reconsideration in contextual affective terms rather than the abstract neurocognitive terms of the model.

#### Efficient Cause

The efficient cause of ADHD would consist of social practices (scientific, clinical, educational, and family) by which certain behaviors of children or adults (material cause) take the form of a diagnostic category (formal cause). The efficient cause has to do rather with the actions of “actors” (Pérez-Álvarez et al., 2008), than with risk “factors” as usually understood (Killeen et al., 2012). Although clinicians are the main “makers” of diagnoses, they are neither the only *agents* nor the first. ADHD *agency* begins in school and family. But clinicians, parents and educators have scientific institutions of reference, such as the *National Institute of Mental Health* (NIMH) in the USA and the *National Institute for Health and Clinical Excellence* (NICE) in the United Kingdom, and associations such as *Children and Adults with Attention-Deficit/Hyperactivity Disorder* (CHADD) which support their practices. These institutes and associations in turn are based on scientific research. So it is really scientific research which molds ADHD (Hawthorne, 2014).

Clinicians epitomize the efficient cause as “official” providers of the diagnosis. Typically, children referred to them by schools are taken to the doctor by their parents and are given the diagnosis and a prescription (Smith, 2013). What happened? The parents referred to the child’s problems which brought them there. The clinician (DSM in hand or in mind) asked questions to confirm that the child had the “symptoms” in the description. Other “complementary” tests may also have been applied. Confirmed: the child *did have* ADHD. In fact, those behaviors really do exist and are observable. The child is from now on observed and defined by ADHD behaviors. Everything else, other behavior, circumstances, contexts or history, remain outside of the description. The clinician believes he has *described* an objective reality, but he has also *created* it this way by selecting some behaviors in detriment to others and elevating them to the category of “symptoms.”

There could be a sort of *Charcot effect* here (Pérez-Álvarez and García-Montes, 2007) by which the clinician “generates” the reality he describes to the extent that the subjects end up by seeing themselves according to the diagnosis. This *looping effect* described by Ian Hacking consists of patients internalizing the biomedical view of their diagnosis (Hawthorne, 2014, pp. 160–161). The confirmation the clinicians receive from their patients should not be taken as proof of the objectivity of the diagnosis. There is nothing more objective in psychiatry than the *grande hystérie* (“major hysteria attack”) described by Charcot (Didi-Huberman, 2004) and which he himself was really molding with his descriptions, drawings and photographs (the “neuroimaging” of the time). Once the category has been created, it works like an “*a priori* category” of the clinician’s understanding, who through his actions (interviews, tests) reinfluences the patient’s understanding, adopting the explanations and definitions offered. The creation of the category itself was already a process of “selection” of the most conspicuous and operative symptoms to which the problem and the individual (decontextualized from his history and circumstances) are reduced, as the Charcot effect suggests (Pérez-Álvarez and García-Montes, 2007). Now each case confirms the category and clinical conviction and at the same time is molded to it as the patient adopts the clinicians point of view if in fact he does not already have it as a “cultural idiom,” such as ADHD usually is. Within a dynamic process, the looping effect reaffirms category and clinician and case and patient or user.

The school itself professes the biomedical conception. As already occurred in the origins of ADHD starting at the end of the 1950s with the figure of the school counselor as the intermediary between the classroom and the clinic (Smith, 2013), the school staff still acts as a bridge. Textbooks used to train special education teachers show a strongly biomedical view (Freedman, 2016). Families also tend to see diverse problems even without the ADHD diagnosis, canceling out other possibilities (Lewis-Morton et al., 2014). The influence, if not “pressure” from the school, along with “information” from parents’ associations and associations of those affected, beginning with the CHADD, end up inculcating ADHD in the family, which the clinician only confirms.

If teachers, parents, and clinicians consult international reference guidelines such as the NIMH and the NICE, they will have the impression of a *consensus* on the biomedical nature of ADHD which really does not exist (Moncrieff and Timimi, 2013; Erlandsson and Punzi, 2016; Erlandsson et al., 2016). All in all, the ultimate or first efficient cause is how science molds the ADHD which then feeds guidelines and their users. As shown by Hawthorne (2014), science molds ADHD the way it is by certain patterns of reasoning (epistemology) and research methods (methodology) which mutually reinforce each other in a continuous dynamic process.

Although there are a variety of approaches, levels of analysis and sciences in the study of ADHD, all of them have two things in common: the object and the method. The object is the DSM-defined ADHD and the method is some version of the “scientific method” (Hawthorne, 2014, pp. 47–48). ADHD is assumed to be a natural, complex entity which must be objectively described and its mechanisms studied. The proper framework implicitly involves the ADHD/no-ADHD dichotomy (it would be stupid to divide the subjects of research repeatedly without thinking that there are no differences among the groups), as well as generalization from statistical means, reasoning linking genetic, neural and behavioral levels, biological reductionism (“mechanisms”) and the final reification of ADHD as an identifiable and treatable species (with its subtypes) (Hawthorne, 2014, p. 70).

The lack of firm evidence is supplemented with rhetoric as already shown above in order to make the reasoning convincing. Thus, “convergence” of a variety of data are discussed even when they are *not* significant and of the need for new more refined studies, giving the impression of being on the right track. As Hawthorne (2014, p. 66) points out, “science builds on previous science; but it is also to say that previous science constrains current science to some extent—novelty is not forbidden, just difficult—by imposing a structure of prior formulation, categorization, and contexts—and tools, techniques, and experimental models—of interest.” In scientific practice, a Charcot effect by which research studies what it generates itself (hypotheses and so forth) in a sort of “collective hysteria” would not be unthinkable.

Summarizing, the efficient cause of ADHD would be found in scientific, clinical, educational, and family practices that *make* it the way it is. Without denying that it is *real*, the efficient cause shows how it *becomes* real. If it were a natural entity, as many medical illnesses are (epilepsy, Parkinson’s, Alzheimer’s), it would make no sense to talk about the efficient cause. But neither because it is not natural is it less real and easier to change. The genome and the brain may be more plastic than the scientific practices themselves with their institutionalization, patterns of reasoning and self-confirmatory methods.

#### Final Cause

The final cause of ADHD refers to a series of functions which it meets for a variety of actors and institutions, beyond the reinforcement of behaviors of those affected (Killeen et al., 2012). This variety of unintentional functions could explain its expansion, as well as the conviction with which it is argued against the “nay-sayers,” in spite of the persistent lack of firm evidence, as reviewed above. In fact, the success of ADHD may be due paradoxically to its *imprecision*: a case of the strength of vague concepts (Löwy, 1992). As this author says, “Imprecise concepts may help to link professional domains and to create alliances between professional groups.” ‘Fuzzy’ terms, continues the author, may last a lifetime and keep functioning (Löwy, 1992, p. 373). This is the case of ADHD.

The “trading zone,” which enables imprecise concepts (Löwy, 1992), has its best expression in ADHD as a “semiotic mediator” as defined by Svend Brinkmann. A semiotic mediator referring to a diagnosis is a symbolic linguistic device with three functions: an explanatory function with regard to the problems experienced, a self-affirming function in the sense that a variety of phenomena appear as “symptoms” and a disclaiming function related to responsibility (Brinkmann, 2014b). Semiotic mediation harmonizes the needs, interests and values that make up the ADHD complex. According to Hawthorne (2010, 2014), the solution is reinforced by a positive feedback loop.

A positive feedback loop incorporates values in concepts, methods and scientific conclusions. As the theme of interest chosen, research begins by establishing the division between ADHD and non-ADHD (ADHD versus “controls,” “normal,” “healthy,” “typical development,” “unaffected”). Based on this dichotomy, an infinity of topics (“variables”) are chosen to observe possible neurocognitive correlates and genetic and behavioral associations. When a “difference” is found, as Hawthorne says, “the observed difference is only relatively value-free, having been arrived at through several value-valenced choices. The slip from “difference” to “dysfunction,” which is an ethical term, intensifies the valuation.” Positive results reinforce the decisions and in any case, as they say, more studies are necessary, strengthening the feedback loop (Hawthorne, 2014, p. 136). “Overall, then, the social/scientific feedback loop is self-reinforcing as long as science achieves results that society can take up and support. By this ongoing mutual influence, facts and values are jointly defined and reinforced” (Hawthorne, 2010, p. 28).

Attention-Deficit/Hyperactivity Disorder harmonizes a variety of scientific, medical, educational and family interests besides pharmaceutical industry profits (the most openly shameless and rightly denounced). The only party harmed seems to be the children, with the unintentional effect of “accidental intolerance” of the traits and ADHD-associated behaviors (Hawthorne, 2014, p.142).

Briefly, the final cause of ADHD would be in harmonizing the interests of a variety of actors and institutions, not just the pharmaceutical industry. The particular feedback loop between science and society in which normative values become naturalized and legitimated in research, which is valued and supported by society, has been shown. Perhaps the children are the least benefited due to the resulting “accidental intolerance.” Individual differences become dysfunctions, disorder or mental illness.

## Conclusion

An argument has been developed in three steps. First, the evidence claimed which sustains ADHD was reviewed. It was shown that the diagnosis is based on fallacious reasoning (tautologies), for lack of clinical proof. At this point, the lack of specific genetic and neurobiological evidence should not be surprising, in spite of the enormous amount of literature pointing in that direction. The fact that the science of ADHD seems to be going on the right path is probably due more to the rhetoric and metaphysics of its literature than to accumulated scientific findings. Rhetoric in use seems to convert ambiguous meager data into conclusions presented as “convergent evidence,” where the overused expressions “complex” or “heterogeneous” disorder really means that its causes are unknown, even if assumed to be genetic and neurobiological. Implicit metaphysical assumptions are found for example, in correlates and correlations taken as neural “causes” or “bases.”

Second, a new metascientific approach to the science of ADHD was proposed, supposing that a mere critical review leaves the controversy between “defenders” and “critics” the same as it was, in a dialog of the deaf. More so, criticism, as demolishing as it is, still recognizes the sense, persuasion, and good faith, not ignorance or simple interests, of the defenders. How are they not going to be convinced if the science they profess directs their path, sheds light on the subject and offers the method? The fact is that the science itself may have blind points and self-confirming methods, without even going down the right path. The new metascientific approach proposed is Aristotle’s four causes, material, formal, efficient, and final, as an instrument of enquiry.

Third, the enquiry was carried out using Aristotle’s four causes. In addition to a clarifying view, this metascientific focus offers an alternative to the understanding of “ADHD” beyond the dominant biomedical model and its simple denial. According to this analysis, the *material* of which ADHD is made would be some specific behaviors which can be problematic in certain contexts and tasks. These behaviors are easily taken as the *form* of the “ADHD” diagnostic category according to diagnostic criteria in use (typically the DSM). The *efficient cause* with regard to what, or better, who *makes* ADHD the way it is may be found on one hand in the clinicians who make the diagnosis and on the other in scientific research which has molded the established concept. The *final cause* refers to a variety of functions which ADHD meets, *not* in spite of its scientific-clinical vagueness. On the contrary, it would be precisely due to its *imprecision* which makes it useful in a variety of contexts. The positive science-society feedback loop which surreptitiously combines facts and values as the reason for its success in harmonizing varied interests has been shown.

The metascientific perspective makes it possible to see and go beyond the scientific controversy run aground on whether ADHD exists or not. According to this analysis, ADHD would not be sustainable as a clinical entity, although it is still real as a practical entity (Pérez-Álvarez et al., 2008). Far from being a given *natural kind*, out there, ready for its research as a scientific *object*, “ADHD” would be a *practical kind*, constructed on the scientific and clinical practices themselves, fulfilling a variety of functions, who knows for whom or at what cost. Beyond the positivist science framework (typically neuroscience), the “number one recommendation” would be to “establish a pragmatist framework that carefully uses facts *and* values in all decisions and actions relevant to ADHD” (Hawthorne, 2014, p. 176). As a scientifically and ethically coherent derivation, the “ADHD”/non-“ADHD” dichotomy would have to be overcome, and instead of the *essentialist* conception in use, adopt a pragmatic approach with regard to situations and concrete norms where the problem can be enacted according to a cultural, contextual, existential psychology (Brinkmann, 2016, p. 88).

Therefore, the problems to which “ADHD” refers should never have left the family and school educational scope, making them pass through the clinical circuit and come back as “mental disorder.” Any problems related to “attention,” “activity,” and “impulsivity” are not outside learning as aspects of development of self-control. Some children may require additional “training” (not treatment). More precisely, such “training” would be by parents and teachers with a view to promoting the skills children require. Training by parents using common games involving attention and following rules (“Simon says,” “frozen dance”) as well as behavioral principles (availability of appropriate contexts, positive reinforcement), would “remove” children from (risk of receiving the diagnoses of) “ADHD” (Charach et al., 2013; Laber-Warren, 2014). A study with a careful design showed that behavioral training by parents and teachers was more effective than medication (Pelham et al., 2016). These behavioral “interventions,” more than as an alternative to medication (which is no small thing), are referred to here as an ontological argument demonstrating the practical behavioral nature (non-essentialist) of ADHD. It is already time to overcome the “ADHD”/non-“ADHD” dichotomy without paying attention to the aspect of the problem when necessary without pathologizing it. Since the diagnostic language is not inevitable, only dominant,

We must supplement the pragmatic interest in action possibilities [afforded by different languages inherent in social practices (e.g., existential, moral, political)] with a hermeneutic interest in interpreting the person and her suffering in her life situation as it presents itself in its “facticity” (Brinkmann, 2014a, p. 645).

## Author Contributions

The author himself conceived, wrote, reviewed and approved the article.

## Conflict of Interest Statement

The author declares that the research was conducted in the absence of any commercial or financial relationships that could be construed as a potential conflict of interest.
